# l‐Dopa/carbidopa intestinal gel and subthalamic nucleus stimulation: Effects on cognition and behavior

**DOI:** 10.1002/brb3.848

**Published:** 2017-10-20

**Authors:** Francesc Valldeoriola, Pilar Santacruz, José Ríos, Yaroslau Compta, Jordi Rumià, José Esteban Muñoz, María José Martí, Eduardo Tolosa

**Affiliations:** ^1^ Parkinson and Movement Disorders Unit Neurology Service Institut Clínic de Neurociències Hospital Clínic Barcelona Spain; ^2^ Institut d'Investigacions Biomèdiques August Pi I Sunyer (IDIBAPS) University of Barcelona Barcelona Spain; ^3^ Parkinson and Movement Disorders Unit ‐ Neurology Service Fundació Clínic per la Recerca Biomèdica Barcelona Spain; ^4^ Medical Statistics Core Facility IDIBAPS (Hospital Clinic) Barcelona Spain; ^5^ Biostatistics Unit Faculty of Medicine Universitat Autònoma de Barcelona Barcelona Spain; ^6^ Neurosurgery Service Institut Clínic de Neurociències Hospital Clínic Barcelona Spain; ^7^ Department of Surgery and Surgical Specialties University of Barcelona Barcelona Spain

**Keywords:** behavior, cognition, intestinal l‐dopa, Parkinson, subthalamic stimulation

## Abstract

**Objective:**

In Parkinson's disease (PD), effects on behavior and cognition of levodopa/carbidopa intestinal gel (LCIG) and subthalamic stimulation (STN‐DBS) and their practical consequences remain controversial. This study was designed to analyze the possible effects of these therapies on cognition and behavior after 1 year follow‐up.

**Methods:**

This was an open‐label, nonrandomized prospective study for pre‐ and postintervention analyses. Twenty‐four patients were considered eligible to be candidates for complex therapies such as STN‐DBS or LCIG; 23 patients treated with standard medication were included as controls. Several cognitive, behavioral, and motor scales were administered before and at 6 and 12 months after the intervention.

**Results:**

Patients treated with LCIG experienced significant improvement in specific neuropsychological functions when compared with patients receiving STN‐DBS and conventional medical treatment after 1 year from the onset of the intervention. In this study, no significant cognitive or behavioral changes occurred in patients treated with subthalamic stimulation when compared to patients receiving conventional medical treatment at 1 year follow‐up.

**Conclusions:**

Patients treated with LCIG may significantly improve some specific neuropsychological functions when compared with patients receiving STN‐DBS and with patients receiving conventional medical treatment after 1 year from the intervention; there are not significant cognitive or behavioral changes in patients treated with STN‐DBS when compared to PD patients receiving conventional medical treatment after 1 year from the intervention. The outcomes showed in the study can help to the selection of the appropriate candidates for STN‐DBS and LCIG.

## 1 INTRODUCTION

Levodopa/carbidopa intestinal gel (LCIG) and subthalamic nucleus deep brain stimulation (STN‐DBS) are complex treatments for patients with advanced Parkinson's disease (PD), refractory to conventional treatments. Effects of STN‐DBS in cognition and behavior have been analyzed in many studies. The effects of STN‐DBS have been studied with findings ranging from no significant changes in cognition and behavior to mild increase in anxiety and worsening of some cognitive functions (Alegret et al., 2001; Saint‐Cyr, Trépanier, Kumar, Lozano, & Lang, 2000). Some studies found mild improvement in mood such as anxiety and depressive symptoms, but no changes in cognitive performance (Witt et al., 2008). Comparative studies revealed equivalent positive subjective and mood‐related effects (Funkiewiez et al., 2004). Assessment of turning on the stimulator improved executive function, but worsened conditional associative and visual conditional learning (Jahanshahi et al., 2000; Pillon et al., 2000). A study comparing the effects on cognition of STN‐DBS and subcutaneous continuous infusion of apomorphine (APM‐CSI) showed that, contrary to APM‐CSI, STN‐DBS produced a worsening in executive functions (Alegret et al., 2004). Functional neuroimaging studies showed that frontal tasks either did not recover or worsened after STN‐DBS over time (Kalbe et al., 2009).

Effects of LCIG on cognition and behavior are not well known, but up to 41% of LCIG‐treated patients showed impaired memory and cognitive flexibility after 3 years follow‐up (Zibetti et al., 2013).

We have studied a group of patients with advanced PD, candidates for LCIG and STN‐DBS in order to compare the effects of these therapies on cognition and behavior. Results at 1 year were also compared with those in a control group of patients treated with standard oral medication during the same period.

## 2 MATERIAL AND METHODS

### 2.1 Study design and subjects

This was an open‐label, nonrandomized prospective study for pre‐ and postintervention analyses. The study was approved by the Hospital Ethics Committee (2009/5222). All patients included in the study signed an informed consent.

Neurologists from the Movement Disorders Unit at the Hospital Clinic enrolled patients aged 50–70 years experiencing disabling motor fluctuations while receiving l‐dopa and other dopaminergic treatments. Eligible patients were those considered candidates for complex therapies such as DBS or LCIG. All patients had idiopathic PD; a Hoehn & Yahr (HY) stage III‐IV during the off period; and disabling motor fluctuations that poorly responded to standard medical therapy. Patients with evidence of dementia, major psychiatric illnesses, and/or progressive medical illnesses were excluded. After initial evaluation, 24 patients were considered candidates for either LCIG or STN‐DBS therapy following current accepted guidelines (Kulisevsky et al., 2013). After detailed explanation of both therapies, patients were assigned to undergo LCIG or STN‐DBS based on patients preference. Some of these patients were not considered candidates for one of these therapies and were enrolled as controls. Reasons for not entering the treatment arm of the study were as follows: patients declined interventions, presence of residual symptoms in on phase that produced significant disability, motor complications were considered to be managed medically, and presence of comorbidity. These patients were matched with those receiving STN‐DBS and LCIG for age, gender, HY stage, disease duration, educational level, and cognition. Four of the patients included in the control group underwent STN‐DBS and other four received LCIG after study completion because of worsening of motor complications.

### 2.2 Therapeutic interventions

Bilateral STN‐DBS was done under standard conditions through stereotaxy and conscious sedation. An stereotactic planning software (iPlan from BrainLab AG, Munich, Germany) was used to determine coordinates of the STN, based on direct targeting in 3 Tesla MRI. Intraoperative microrecording and stimulation were used to confirm an adequate location before implanting the electrode (Model 3389‐40, Medtronic, Minneapolis, MN), extension wires (7495‐51 cm, Medtronic, Minneapolis, MN), and neurostimulator (Kinetra^®^ Model 7428, Medtronic, Minneapolis, MN). Electrical settings and medication were adjusted to an optimal clinical response.

Patients selected for LCIG had a testing period with a nasoduodenal tube and LCIG infusion with Duodopa^®^ (Abbott Products GmbH, Neustadt, Germany). Patients were initially switched overnight from their conventional therapy to LCIG. A percutaneous endoscopic gastrostomy (PEG) with jejunal tube (Fresenius Kabi AG, Bad Homburg, Germany) was indicated in those patients presenting good clinical response to the treatment; l‐dopa/carbidopa via PEG was titrated according to patient's need during ensuing weeks. Instructions for stoma care and managing of the infusion pump (CADD‐Legacy Model 1400, Smiths Medical ASD Inc., St. Paul, MN, USA) were given by a nurse.

### 2.3 Cognitive assessment

Cognitive assessment was done by the same neuropsychologist. Basal testing was done within a month prior the procedure. Patients were also evaluated at 6 and 12 months after the intervention.

We used MMSE (Mini‐Mental State Examination) and the Scales for Outcomes in Parkinson' disease cognition (SCOPA‐Cog) screening to assess if patients had dementia. Cut‐off scores were ≥24 for MMSE and ≥19 for SCOPA‐Cog. All scales used were standardized by age and years of schooling according to the Spanish population. Posttreatment evaluation and final assessment were programmed at 6 and 12 months after interventions. All patients were assessed in on phase with the same timetable.

The Word List Learning Test from the Wechsler Memory Scale Third Edition (WMS‐III) was used to assess verbal learning, delayed recall, and recognition memory.

Executive function was assessed by semantic fluency (animals in 1 min), verbal fluency (FAS test), and the Stroop test (word, color, and word–color subtests) for the phonological category, tests that measure spontaneous production of words belonging to the same category or beginning with some designated letter. Also, we used the Stroop words, colors, and interference test. Visuospatial ability was examined by the Benton Judgment of Line Orientation test (BJLO).

### 2.4 Mood and behavior assessment

Depressive symptoms were assessed by the Beck's Depression Inventory (BDI‐II) and apathy by the Lille Apathy Rating Scale (LARS). The behavioral construct of impulsiveness was measured by the Barratt Impulsiveness Scale (BIS‐30) and Maudsley Obsessional–Compulsive Inventory (MOCI).

#### 2.4.1 Fatigue assessment

The Parkinson fatigue scale (PFS) and the multidimensional fatigue scale (MFS) were used to consider fatigue and its impact on the patient's daily function.

#### 2.4.2 Motor evaluation

Motor evaluation was assessed on the same day, then the neuropsychological battery was administered. We used the Unified Parkinson's Disease Rating Scale (UPDRS), H‐Y staging, and Schwab & England Activities of Daily Living Scale (S‐E). The UPDRS assessments were done during “on” period. UPDRS‐IV items 32–33 for dyskinesia and 39 for motor fluctuations were used to measure motor complications.

#### 2.4.3 Medication

Patients on LCIG were given a l‐dopa equivalent dose of 1,145 ± 305 mg at baseline and 1,205 ± 239 mg at final follow‐up (increase 5%). Patients receiving STN‐DBS were given a l‐dopa equivalent dose of 900 ± 275 mg at baseline and 579 ± 281 mg at final follow‐up (reduction 36%); for control group, l‐dopa equivalent dose were 875 ± 347 mg and 897 ± 374 mg at baseline and final follow‐up, respectively (increase 2%).

### 2.5 Statistical analyses

Descriptive data of the three groups were shown as a median with 25th, 75th percentiles for quantitative variables, and absolute frequencies and percentages. All scales used in the present study were standardized by age and years of schooling according to the Spanish population. Analyses of results at follow‐up were presented by estimation of means and 95% confidence intervals (95% CIs) in order to compare differences between groups as main objective, from a general lineal model (GLM). This analysis included group (main analyses factor), time, and their interaction as factors and baseline results of dependent variable as covariable. All data were analyzed with statistical analysis software (SPSS version 20, Armonk, NY: IBM Corp) and values of *p *≤ .05 were considered statistically significant. Due to methodological characteristics of this study, the *p*‐values presented were nominal and were not adjusted for multiplicity.

## 3 RESULTS

### 3.1 Baseline assessment

Demographic and clinical data of subjects at baseline are shown in Table 1. No significant differences in these parameters were found between groups. As previously described, all patients had a MMSE ≥24 and a SCOPA‐cog ≥19 to enter the study; however, we found significant differences between groups in:

**Table 1:** Basal demographic and clinical data of the patients of the study

	LCIG (*N *= 11)	STN‐DBS (*N *= 12)	Control patients (*N *= 23)
Male	8 (72.7%)	11 (91.7%)	19 (82.6%)
Age	64 [59, 72]	57 [51, 63]	63 [55, 65]
Years of schooling	8 [8, 10]	11 [10, 15]	10 [8, 14]
Hoehn and Yahr (on)	2.5 [2.5, 2.5]	2.3 [2, 2.5]	2 [2, 2.5]
UPDRS‐I	2 [0, 3]	2 [0, 2]	1 [0, 2]
UPDRS‐II (on)	8 [6, 14]	9 [7, 11]	8 [6, 8]
UPDRS‐III (on)	22 [14, 23]	14 [10, 22]	14 [7, 23]
UPDRS‐IV	6 (3) 7 [4, 9]	6 (4) 6 [4, 8]	3 (3) 1 [0, 5]
Schwab & England (on)	80 [80–80] (70–90)	80 [80–90] (80–90)	90 [80–90] (50–100)
Disease duration	14.5	13	12
l‐Dopa equivalent dose (mg)	1,145 (305)	900 (275)	875 (347)
MMSE	28 [27, 29] (24–30)	29 [28, 29] (26–30)	28 [27, 29] (24–30)
MMSE ≥24	11 (100%)	12 (100%)	23 (100%)
SCOPA‐Cog	25 [24, 29] (19–33)	27 [25, 29] (22–31)	27 [24, 29] (20–34)

LCIG, levodopa/carbidopa intestinal gel; STN‐DBS, bilateral subthalamic deep brain stimulation.

Data are represented as median [25th, 75th percentiles] for qualitative variables and as an absolute frequency and percentage for quantitative variables. For Schwab & England, MMSE, and SCOPA‐cog data, the numbers in the lower row are the minimum and maximum scores.

*Learning and recall:* LCIG patients scored worse than patients who underwent DBS both in WMS delayed recall (2.45 [1.01, 3.89], *p* < .01) and recognition (1.11 [0.64, 1.59], *p *< .001); similarly, LCIG patients scored worse than controls in WMS recognition (1.1 [0.73, 1.48], *p *< .01).

*Frontal functions:* Stroop word was better performed by STN‐DBS (−.61 [−1.02, −0.2], *p *= .003) and CDLI (−.45 [−.68, −.22], *p *< .001) candidates than by control patients.

*Mood and behavior:* MOCI scores were lower in STN‐DBS patients than controls (1.37 [0.31, 2.44], *p *= .01); by contrast, BDI‐II showed worse scores in STN‐DBS (1.9 [0.57, 3.23], *p *= .005) and LCIG candidates (2.86 [1.01, 4.71], *p *= .002) than controls. CDLI patients showed higher scores in LARS than in STN‐DBS patients (3.52 [1.63, 5.41], *p *< .001) and controls (3.65 [1.54, 5.76], *p *< .01).

*Fatigue:* MFS showed significant worse scores in patients that were candidates to both interventions than in control subjects, LCIG (5.43 [1.78, 9.08], *p *= .003); STN‐DBS (4.29 [0.16, 8.43], *p *= .04).

### 3.2 Intergroup comparisons at follow‐up

*MMSE:* No significant differences were found between any group of patients.

*Scopa‐Cog:* As per inclusion criteria, all patients had a Scopa‐cog score ≥19. Yet, patients selected for LCIG scored significantly lower than STN‐DBS patients at basal assessment (−1.85 [−3.28, −.42], *p *= .01) and STN‐DBS patients had significant better scores than controls (2.1 [.66, 3.53], *p *= .004). By contrast, LCIG patients improved Scopa‐cog scores both at 6 (−1.77 [−3.14, −.39], *p *= .1) and 12 months (−3.13 [−5.36, −.89], *p *= .006) follow‐up compared with controls. Furthermore, significant differences observed between control patients and STN‐DBS patients and between STN‐DBS and LCIG patients at baseline disappeared at follow‐up.

#### 3.2.1 Learning and recall

*WMS learning:* LCIG patients scores significantly improved with respect to STN‐DBS patients (−3.2 [−6.34, −.07], *p *= .04) and control subjects (−3.52 [−5.73, −1.31], *p *= .001) at 6 months and at 1 year follow‐up (−2.64 [−5.24, −.05], *p *= .04).

*WMS delayed recall:* LCIG patients significantly improved with respect to STN‐DBS patients (−3.36 [−6.7, −.01], *p *= .04) and controls (−3.97 [−6.54, −1.4], *p *= .002) at 6 months follow‐up, and with respect to STN‐DBS patients (−3.08 [−6.04, −.12], *p *= .04) and control patients (−3.77 [−5.92, −1.61], *p *< .001) at 1 year follow‐up.

**Figure 1:**
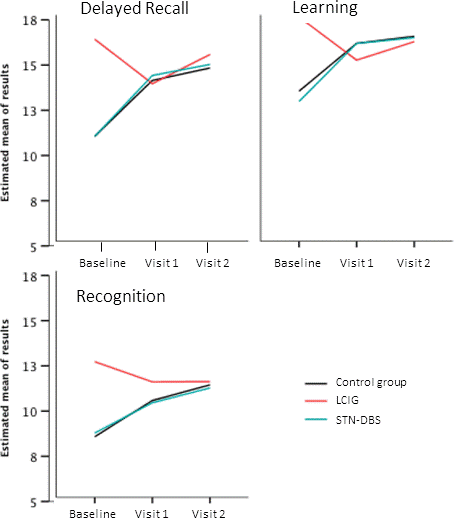
Performance scales standardized for Spanish population scores on the Word List Learning Test of the WMS‐III at baseline and 6 and 12 months follow‐up

*WMS recognition:* LCIG patients significantly improved with respect to STN‐DBS patients (−1.67 [−3.2, −.13], *p *= .03) and control subjects (−2.49 [−3.91, −1.07], *p *< .001) at 6 months follow‐up, and at 1 year follow‐up, (−2.00 [−3.74, −.25], *p *= .025) regarding STN‐DBS patients and (−2.86 [−4.48, −1.24], *p *< .001) with respect to control patients (Figure 1).

**Figure 2:**
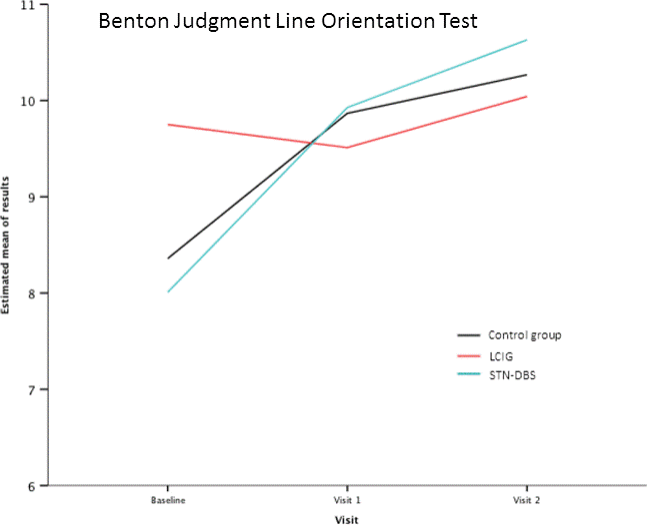
Results obtained in the Benton Judgment of Line Orientation test by the three groups of patients at the baseline and after follow‐up

*Visuospatial ability:* Significant improvement of BJLO test scores was observed in patients treated with LCIG (−1.92 [−3.2, −.64], *p *= .003) compared to those treated with STN‐DBS at 6 months and at 1 year (−1.51 [−2.87, −.15], *p *= .02), and compared with controls at 6 months (−2.62 [−3.94, −131], *p *= .0001) and 1 year (−1.91 [−3.1, −.71], *p *= .001) (Figure 2).

**Figure 3:**
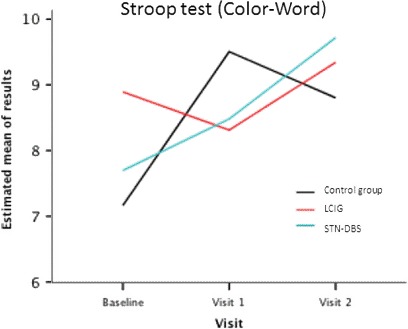
Results obtained in the Stroop test (color–word) by the three groups of patients at the baseline and final follow‐up

*Frontal functions:* LCIG patients mildly improved performance of Color–Word of Stroop test at final follow‐up compared with the STN‐DBS group (−2.25 [−4.47, −.04], *p *= .04) (Figure 3).

*Mood and behavior:* No significant changes between groups were found in BDI‐II, LARS, BIS, or MOCI. However, basal differences found in LARS and MOCI with respect to controls (worse scores in LCIG patients) disappeared.

*Fatigue:* No significant changes between groups were found in PFS and MFS during the study period compared to baseline scores.

Patients treated with STN‐DBS did not show any significant differences in any of the neuropsychological and behavioral assessments in relation to control patients at follow‐up evaluations.

### 3.3 Motor assessment

#### 3.3.1 Baseline evaluation

Statistical analyses revealed some intergroup differences in basal evaluation (“on” condition) that could be understood giving the naturalistic design of the study. LCIG patients scored worse than controls in UPDRS‐I (0.27 [0.01, 0.54], *p *= .04). However LCIG patients showed better scores in UPDRS‐II with respect to STN‐DBS patients (0.69 [−1.3, −.08], *p *= .02) and STN‐DBS patients presented worse UPDRS‐II scores than control patients (1.11 [.57, 1.66], *p *= .0001). With respect to UPDRS‐III, LCIG patients presented higher scores than controls (2.18 [.88, 3.47], *p *= .001). Both LCIG (1.97 [1.29, 2.65], *p *< .0001) and STN‐DBS (1.39 [0.5, 2.28], *p *= .002) patients had significantly higher UPDRS‐IV scores than controls. However, we found that both LCIG (−.43 [−1.78, 0.92], *p *= .03) and STN‐DBS (−1.08 [−2.06, −.09], *p *= .03) patients presented better S‐E scores compared to controls.

### 3.4 Follow‐up

At the end of the study a significant improvement was observed in patients on STN‐DBS compared to control patients in UPDRS‐IV item 32 (−1.4 [−1.81, −1], *p *< .001), item 33 (−1.58 [−2.06, −1.09], *p *< .001) for dyskinesia, and item 39 for motor fluctuations (−1.00 [−1.34, −.58], *p *< .001). CDLI patients did also show a significant improvement at final follow‐up compared to controls concerning the item 32 of UPDRS‐IV (−1.03 [−1.50, −.55], *p *< .001), item 33 (−1.12 [−1.55, −.68], *p *< .001), and item 39 (−1.25 [−1.71, −.80], *p *< .001). Such improvements are similar to those observed with this scale in previous studies both with LCIG (Aarsland & Kurz, 2010) and STN‐DBS (Eggert et al., 2008). No significant differences in these measurements were observed between STN‐DBS and LCIG patients.

### 3.5 Side effects and complications during the study period

*LCIG group*: Malfunctioning of the pump occurred in one patient in the CLD group due to impaction of intestinal tube in the intima of the gut and the tip of the tube surrounded by a bezoar. Changing the gastrointestinal tube resolved the problem without complications.

*STN‐DBS group*: One patient developed transient (1 month) hypersexuality after STN‐DBS that resolved after resetting electrical parameters. One patient suffered a car accident due to a sudden sleep episode attributed to treatment with dopamine agonists. Patient needed hospitalization during 2 weeks and recovered without sequels. One patient had transitory suicidal ideation that resolved after psychological help, without need of pharmacological action. One patient had sudden worsening of parkinsonism several months after intervention due to a broken connection. Wire was replaced and the patient returned to previous clinical condition.

*Control patients*: One patient developed edema in legs that was attributed to use of dopamine agonists.

## 4 DISCUSSION

To our knowledge, this is the first study to compare effects on cognition, mood, and behavior of LCIG with those of STN‐DBS and a group of patients on best medical treatment. Patients receiving LCIG improved specific neuropsychological functions such as learning, delayed recall, and recognition of the WMS, Color–Word of Stroop test, and visuospatial function compared to those receiving STN‐DBS and control patients. Improvement in some behavioral aspects such as apathy also occurred in LCIG patients compared with controls. These changes were obtained in the context of significant amelioration in motor fluctuations in both LCIG and STN‐DBS groups.

There is limited information on the effects of LCIG on behavior and cognition; most studies have focused on its effects in motor disability, nonmotor symptoms, and quality of life (Olanow et al., 2014). A study on 17 patients showed that, after LCIG, 41% patients developed cognitive deterioration over time in memory functions, attention, visual‐motor speed, and executive functions (Zibetti et al., 2013), but it could not be excluded that cognitive changes were related to disease progression. Prospective studies have shown that up to 75% PD patients may develop dementia during disease course (Aarsland & Kurz, 2010; Eggert et al., 2008). Patients in which several psychiatric symptoms were prominent before initiation of LCIG showed no worsening of psychosis and improvement of anxiety after the infusion (Sánchez‐Castañeda et al., 2010); moreover, two patients with cognitive impairment were reported to experience a marked improvement after LCIG (Fera et al., 2007).

In our study, cognitive changes in the LCIG group could be related to a positive effect of l‐dopa on some aspects of cognition (Cools, Stefanova, Barker, Robbins, & Owen, 2002; Kulisevsky et al., 2000; Muslimovic, Post, Speelman, & Schmand, 2005). Experimental fMRI studies have shown that l‐dopa administration enhances prefrontal cortex activity, improves cognition in de novo patients and induces blood flow changes in right dorsolateral prefrontal cortex (Poletti et al., 2012). These studies suggest that impairment of executive functions seen in PD from early stages (Cools, Altamirano, & D'Esposito, 2006; Sawamoto et al., 2008) is probably not due to direct pathological derangement of frontal cortex, but due to reduced dopaminergic striatal stimulation, disrupting the functioning of frontostriatal circuits (Poletti & Bonuccelli, 2012; Rowe et al., 2008). Orbital frontostriatal circuits are mostly preserved in early PD, but progressive dopamine depletion would also impair the orbital–frontostriatal circuit (Baunez, Yelnik, & Mallet, 2011).

A second upshot of this study is that patients treated with DBS showed no significant cognitive and behavioral changes with respect to controls at final follow‐up. Variable effects reported after STN‐DBS may be related to the use of different cognitive tasks in the different published studies, small sample of patients, use of concomitant medication, direct effects produced by the intervention, or the expertise of medical teams. There are several anatomical and functional evidences showing involvement of STN in associative and limbic loops (Greenhouse, Gould, Houser, & Aron, 2013), consequently, precise electrode location is crucial according to the functional nonmotor somatotype of the STN (Campbell et al., 2008; Mikos et al., 2011). PET studies suggested that variability in the effects of STN‐DBS on cognitive performance relates to STN‐DBS‐induced cortical blood flow changes in the dorsolateral prefrontal cortex and the anterior cingulate cortex (Zangaglia et al., 2009). Other studies disclosed alteration of phonemic fluency after STN‐DBS compared to control patients (Deuschl et al., 2006). Longer series suggested that STN‐DBS cognitive effects do not influence quality of life (Contarino et al., 2007) or daily living activities (Zangaglia et al., 2012). Long‐term studies have shown that the proportion of STN‐DBS patients who converted to dementia was not different compared to those receiving medication at 2 years follow‐up (Zibetti et al., 2011). Overall, STN‐DBS does not seem to modify the cognitive evolution along the course of the disease (Rodriguez‐Oroz, Obeso, & Lang, 2005; Merola et al., 2011).

Our study was not randomized, and there were between‐groups differences at baseline. Consequently, results have been individually standardized; statistical evaluation of results has been adjusted for baseline data. Conclusions have been made conditional on respect and valuing the fact that, systematically, various neuropsychological analyses eventually converge in the same direction. This fact can give a general idea despite unavoidable methodological problems such as lack of masking of treatment received. Another limitation of the study is that LCIG patients scored worse in several neuropsychological tests at baseline may be in the context of normal clinical practice, since candidates with some cognitive deficits could have been directed to a less aggressive therapy. For the same reason, possibly, patients in the control group who initially declined LCIG or STN‐DBS had less severe motor complications at baseline. In the context of this study it is not possible to evaluate one aspect of patient evolution, which means that there may be a single variable designated as “primary end point.” The fortress of the conclusion must be based on consistency analysis of neuropsychological evolutionary rather than an analysis of one of them in particular.

## 5 CONCLUSIONS

This study shows that some neuropsychological functions may improve in patients with advanced PD treated with LCIG when compared with those receiving STN‐DBS or medical treatment.

No cognitive or behavioral changes were observed in patients treated with STN‐DBS compared to those on medical treatment.

These findings can be of some help to physicians in the selection of candidates for these complex therapies. These conclusions are based in a nonrandomized study with a limited sample size. However, we believe that these patients are highly representative of daily clinical practice in a specialized center. Then, it was not possible to adjust for baseline differences models, which would be advisable in a study not randomized. Despite these baseline differences, there is a convergence of results which give consistency to the findings.

## DISCLOSURE AND CONFLICT OF INTEREST

Francesc Valldeoriola received honoraria from lectures and advice from Abbvie, Medtronic, Boston Scientific, TEVA, Zambon, ItalFármaco, and Sounovion. Jordi Rumià received honoraria for lectures and advice from Boston Scientific and Medtronic. Eduardo Tolosa received honoraria from lectures and advice from Abbvie, Medtronic, Boston Scientific, UCB Pharma, and ItalFármaco. Pilar Santacruz, José Rios, Yaroslau Compta, Ana Cámara, José Esteban Muñoz, and María José Marti have no conflict of interest.
